# Transcription factor KLF4 regulated STAT1 to promote M1 polarization of macrophages in rheumatoid arthritis

**DOI:** 10.18632/aging.204128

**Published:** 2022-06-24

**Authors:** Qiao Ye, Fang Luo, Tingting Yan

**Affiliations:** 1 Department of Rheumatology and Immunology, The Second Affiliated Hospital of Jiaxing University, Jiaxing 314001, Zhejiang, China

**Keywords:** rheumatoid arthritis, transcription factor, KLF4, macrophage, M1 polarization

## Abstract

This study aimed to reveal the mechanism of transcription factor Kruppel-like factor 4 (KLF4) in regulating M1 polarization of macrophages in rheumatoid arthritis (RA) in order to induce inflammatory response. The results suggested that KLF4 overexpression promoted the M1 polarization of RAW 264.7 cells, increased STAT1 expression and up-regulated the phosphorylation level. After KLF4 silencing, the M1 polarization level was down-regulated. Besides, the induced M1 macrophages were co-cultured with articular chondrocytes. KLF4 overexpression further aggravated chondrocyte injury, increased the cell apoptosis rate and activated the inflammatory injury. However, pretreatment with STAT1 inhibitor Cerulomycin resisted the effect of KLF4, and significantly suppressed STAT1 expression and M1 polarization of cells. KLF4 overexpression aggravated synovial tissue injury in mouse joints, up-regulated the expression of inflammatory factors, and increased the levels of CD86 and STAT1.

It was discovered that transcription factor KLF4 promoted the transcription of STAT1 to regulate the M1 polarization of macrophages, thus aggravating the progression of RA and inflammatory response.

## INTRODUCTION

Rheumatoid arthritis (RA) is a kind of multi-system inflammatory autoimmune disease that mainly involves bones and joints [1]. Macrophages have the functions of phagocytosis, chemotaxis and immunoregulation, which participate in specific and non-specific immune responses, and exert a critical role in the genesis and development of RA [2, 3]. Polarization of different macrophage subtypes and its role are the recent hotspots in research on the RA pathogenic mechanism. Macrophages can be mainly divided into the classically activated M1 type and the selectively activated M2 type [4]. The immuno-inflammatory response in RA patients directly influences the polarization of macrophages in peripheral blood, synovium and synovia [5, 6], which increases the number of M1 type pro-inflammatory macrophages, thus breaking the M1/M2 balance [7]. According to macrophage polarization research, the activation of STAT1 signal is directly related to M1 polarization [8] while the transcription regulatory mechanism of STAT1 has not been reported yet.

Kruppel-like factor 4 (KLF4) is a kind of transcription regulatory factor, which can bind to the promoter of target mRNA to stimulate the transcription of mRNA into protein. Some studies have reported the mechanism of KLF4 in tumor genesis and development [8]. It has been found in RA research that KLF4 is associated with the Th17 differentiation in the microenvironment [9] and the inflammatory factor expression in RA [10]. We know that inflammatory factors in RA play an important role in cartilage damage, and previous studies have found that KLF4 is positively correlated with the expression of inflammatory factors [10]. Inflammatory factors are mainly produced by macrophages, especially M1-type cells, so we speculate that KLF4 may be related to the formation of M1-type macrophages in RA. However, the precise mechanism remains unclear. Therefore, the present work aimed to investigate the effect of KLF4 on the polarization of macrophages in RA.

## RESULTS

### Effect of KLF4 overexpression on macrophage polarization

**Figure 1:**
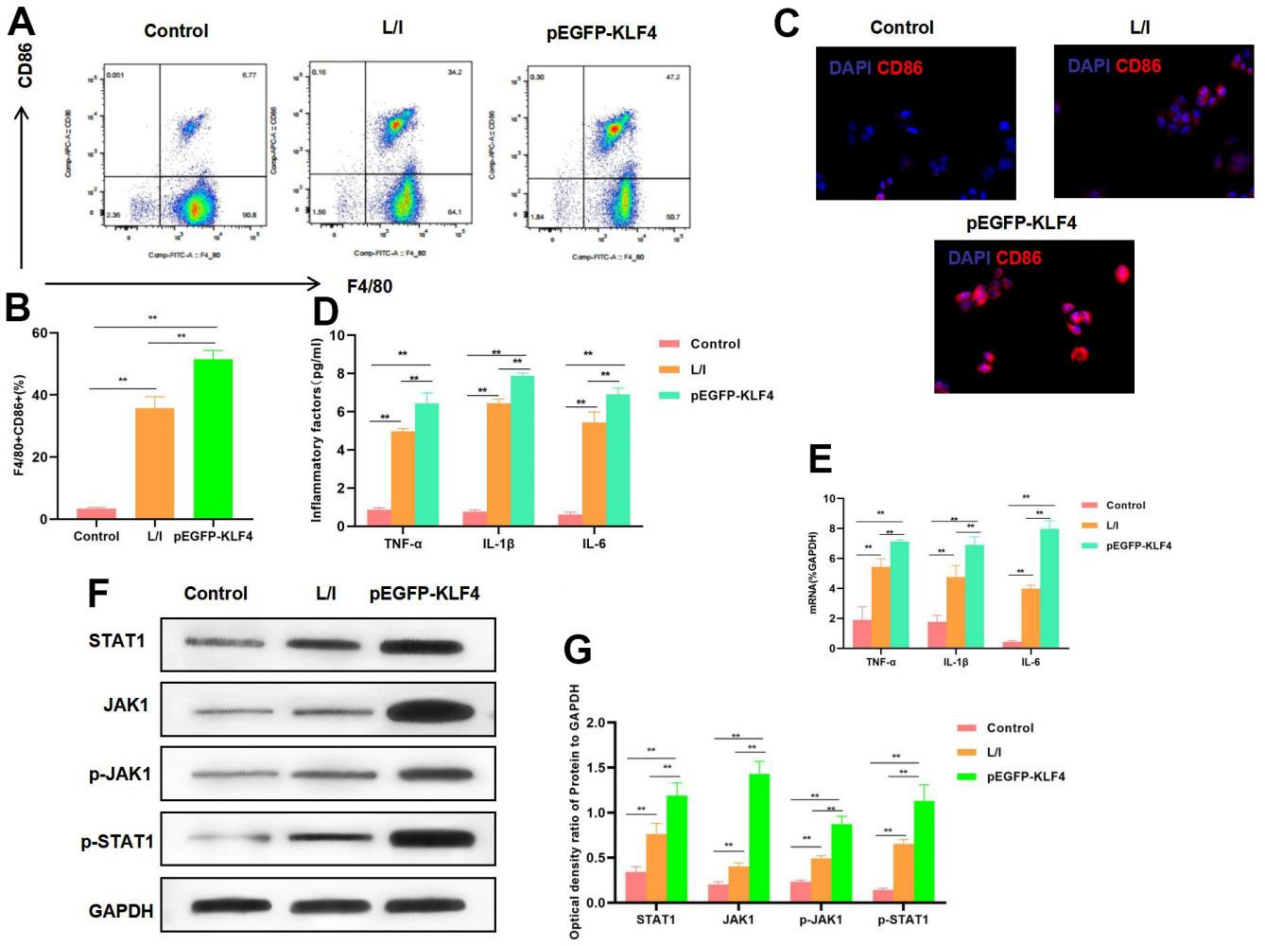
**Effect of KLF4 overexpression on M1 polarization of macrophages.** (**A**, **B**) The proportion of M1 cells was detected by flow cytometry (n=3): KLF4 overexpression (pEGFP-KLF4) promoted the M1 polarization of macrophages and increased the F4/80+CD86+ cell proportion. **P<0.01 between groups. (**C**) IF staining of CD86 expression (n=3). KLF4 overexpression (pEGFP-KLF4) promoted CD86 expression and increased the fluorescence intensity. (**D**) Expression of M1 cell marker proteins (n=3, TNF-α, IL-6, IL-1β). KLF4 overexpression (pEGFP-KLF4) up-regulated the expression of inflammatory factors TNF-α, IL-6 and IL-1β. **P<0.01 between groups. (**E**) Expression of mRNA (n=3, TNF-α, IL-6, IL-1β). KLF4 overexpression (pEGFP-KLF4) up-regulated the expression of mRNA. **P<0.01 between groups. (**F**, **G**) Expression of JAK1-STAT1 signal proteins (n=3). KLF4 overexpression promoted the expression of JAK1 and STAT1 proteins, increased their phosphorylation levels. **P<0.01, relative protein expression between groups.

In RAW 264.7 cells, KLF4 overexpression significantly promoted its M1 polarization level. According to flow cytometry results, the M1 cell proportion increased in pEGFP-KLF4 group, which was higher than that in L/I group (Figure 1A, 1B). Meanwhile, IF staining results indicated that, CD86 expression was up-regulated. CD86 was the marker of M1 macrophages, KLF4 overexpression promoted CD86 expression, and the fluorescence intensity was significantly higher than that in L/I group (Figure 1C). It was discovered from inflammatory factor detection that, KLF4 overexpression promoted the expression of TNF-α, IL-6 and IL-1β. Consistent with mRNA expression, the cytokine levels were significantly up-regulated in pEGFP-KLF4 group. Meanwhile, inflammatory factors were the markers of M1 macrophages (Figure 1D, 1E). Based on protein detection, KLF4 overexpression promoted the activation of JAK1-STAT1 signal, which not only accelerated the expression of JAK1 and STAT1, but also increased their phosphorylation levels (Figure 1F, 1G).

### Effect of KLF4 silencing on macrophage polarization

**Figure 2:**
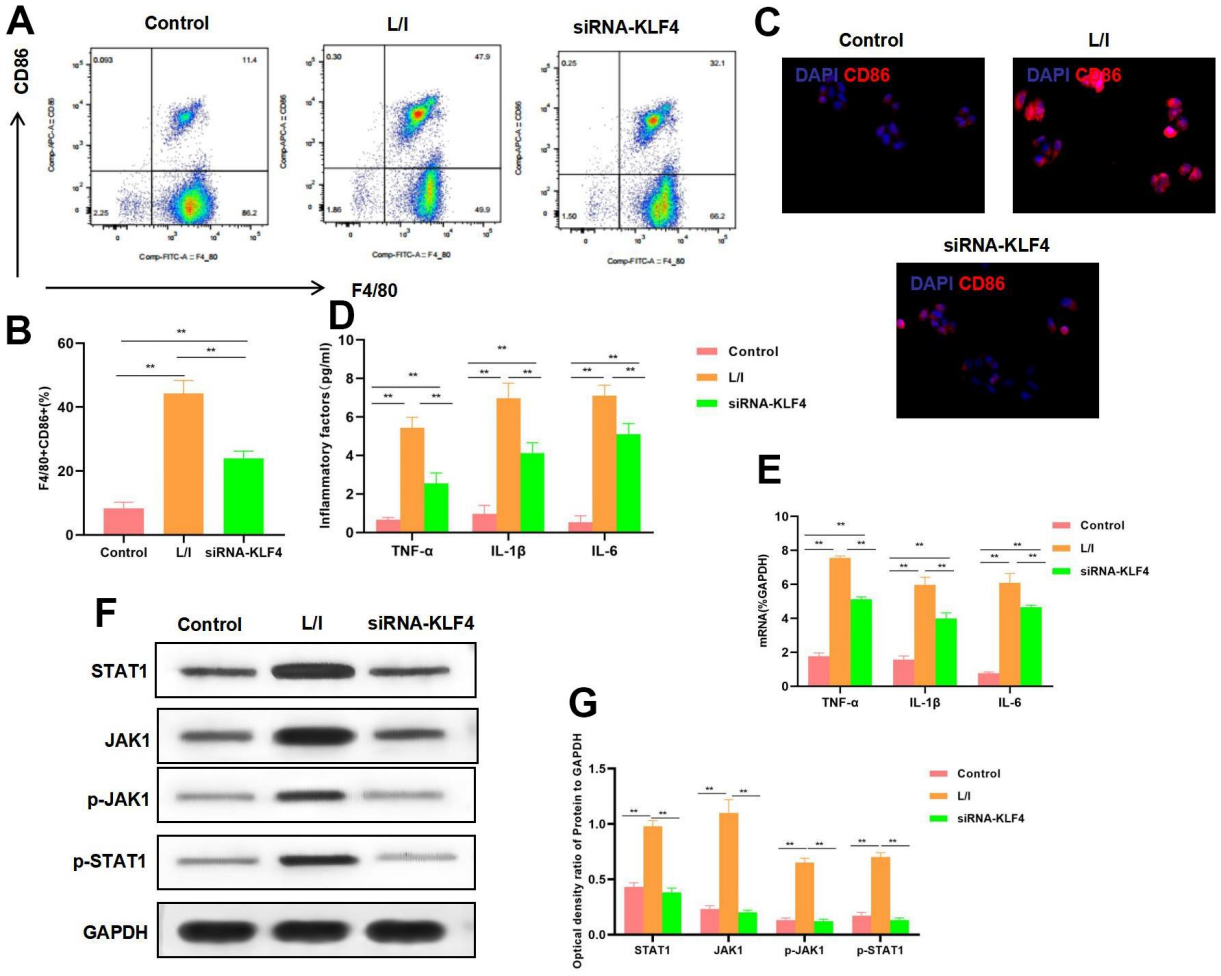
**Effect of KLF4 silencing on M1 polarization of macrophages.** (**A**, **B**) The proportion of M1 cells was detected by flow cytometry (n=3): KLF4 silencing (siRNA-KLF4) decreased the F4/80+CD86+ cell proportion, The proportion of cells in siRNA-KLF4 was lower than that in L/I. **P<0.01 between groups. (**C**) IF staining of CD86. KLF4 silencing suppressed CD86 expression, and decreased the fluorescence intensity. (**D**) Expression of M1 cell marker proteins (n=3, TNF-α, IL-6, IL-1β). KLF4 silencing (siRNA-KLF4) suppressed the expression of inflammatory factors TNF-α, IL-6 and IL-1β. Compared with L/I, the expression of cytokines decreased significantly**P<0.01 between groups. (**E**) Expression of mRNA (n=3, TNF-α, IL-6, IL-1β). KLF4 silencing (siRNA-KLF4) suppressed the expression of mRNA. Compared with L/I, the expression of cytokines decreased significantly**P<0.01 between groups. (**F**, **G**) Expression of JAK1-STAT1 signal proteins (n=3). KLF4 silencing suppressed the expression of JAK1 and STAT1 proteins, decreased the phosphorylation levels. **P<0.01, relative protein expression between groups. There was no difference between Control and BsiRNA-KLF4, but siRNA-KLF4 was significantly lower than L/I.

KLF4 expression was silenced by siRNA. The results suggested that, M1 polarization of macrophages was suppressed, and the proportion of F4/80+CD86+ cells decreased, markedly lower than that in L/I group (Figure 2A, 2B). IF staining results indicated that, CD86 expression was down-regulated, and KLF4 silencing suppressed CD86 expression, with remarkably lower fluorescence intensity than that of L/I group (Figure 2C). The expression of M1 macrophage markers TNF-α, IL-6 and IL-1β was significantly down-regulated, and cytokine expression was consistent with mRNA expression (Figure 2D, 2E). According to protein detection, KLF4 silencing suppressed the activation of JAK1-STAT1 signal, which not only suppressed the expression of JAK1 and STAT1, but also inhibited their phosphorylation levels (Figure 2F, 2G).

### Effect of STAT1 inhibitor pretreatment on KLF4 overexpression

**Figure 3:**
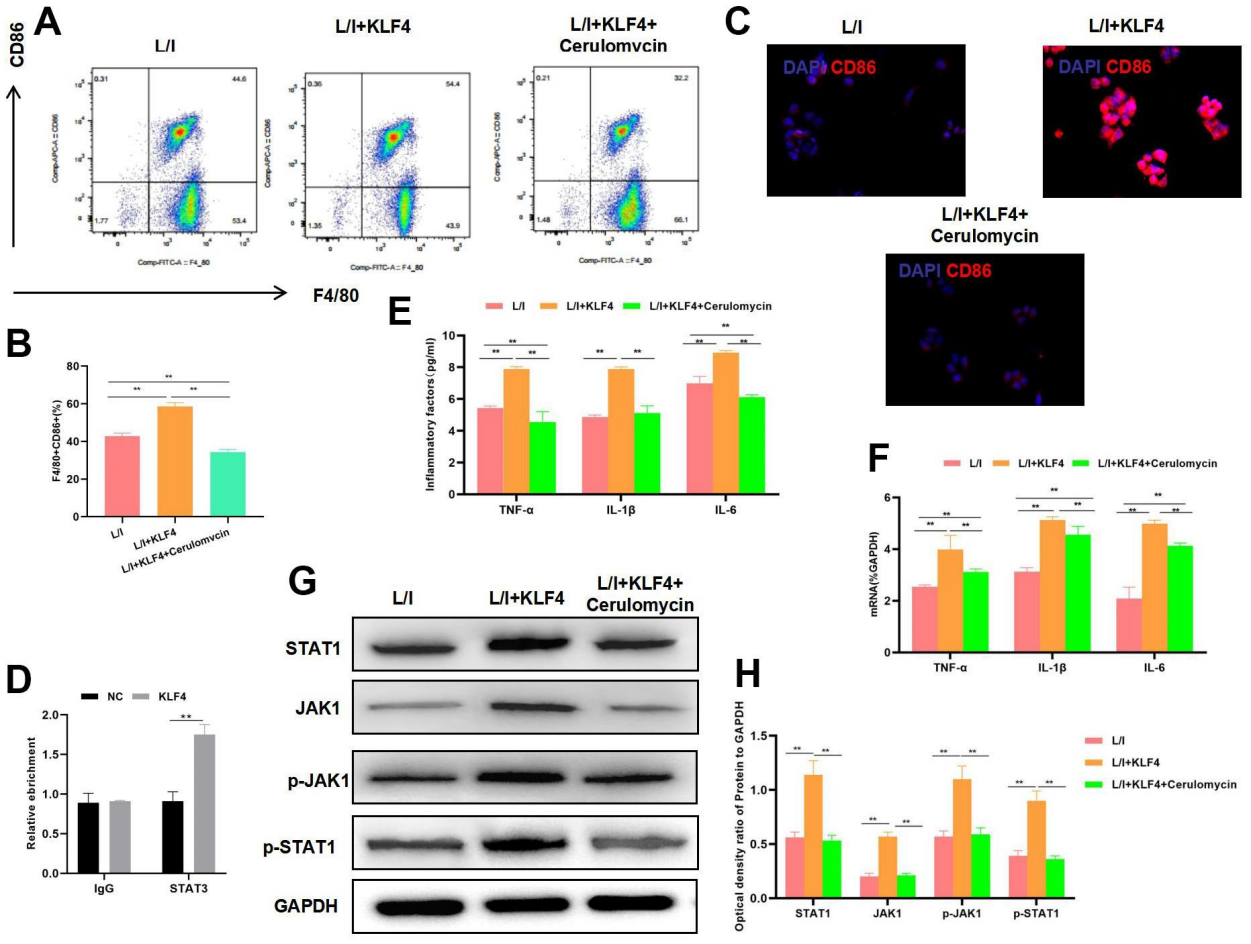
**Effect of STAT1 inhibitor pretreatment on KLF4 overexpression.** (**A**, **B**) The proportion of M1 cells was detected by flow cytometry (n=3): STAT1 inhibitor suppressed the M1 polarization of macrophages and decreased the cell proportion.**P<0.01 between groups. The proportion of M1 cells in L/I+KLF4+Cerulomvcin was lower than that in group L/I+KLF4. (**C**) IF staining of CD86 expression (n=3): STAT1 inhibitor suppressed CD86 expression and markedly decreased the fluorescence intensity. (**D**) RIP assay indicated the binding relation between KLF4 and STAT1. (**E**) Expression of M1 cell marker proteins (n=3, TNF-α, IL-6, IL-1β). After STAT1 inhibitor pretreatment, the expression of inflammatory factors TNF-α, IL-6 and IL-1β was down-regulated. **P<0.01 between groups. L/I+KLF4+Cerulomvcin was lower than that in group L/I+KLF4. (**F**) Expression of mRNA (n=3, TNF-α, IL-6, IL-1β). After STAT1 inhibitor pretreatment, the expression of mRNA was down-regulated. **P<0.01 between groups. L/I+KLF4+Cerulomvcin was lower than that in group L/I+KLF4. (**G**, **H**) Expression of JAK1-STAT1 signal proteins (n=3). After STAT1 inhibitor pretreatment, the expression of JAK1 and STAT1 proteins decreased, and their phosphorylation levels were down-regulated. **P<0.01 between groups.

To verify whether KLF4 exerted its effect through STAT1 transcription, we treated KLF4 overexpression cells with STAT1 inhibitor. The results suggested that, STAT1 inhibitor decreased the M1 cell proportion (Figure 3A). At the same time, IF staining results revealed the down-regulation of CD86 expression and decreased fluorescence intensity (Figure 3B). RIP assay indicated that KLF4 specifically bound to STAT1 (Figure 3C), the expression of M1 cell markers TNF-α, IL-6 and IL-1β was significantly down-regulated, and cytokine expression was consistent with mRNA expression (Figure 3D, 3E). Treatment with STAT1 inhibitor suppressed the activation of JAK1 signal and its phosphorylation level (Figure 3F, 3G).

### M1 macrophages induced chondrocyte injury

**Figure 4:**
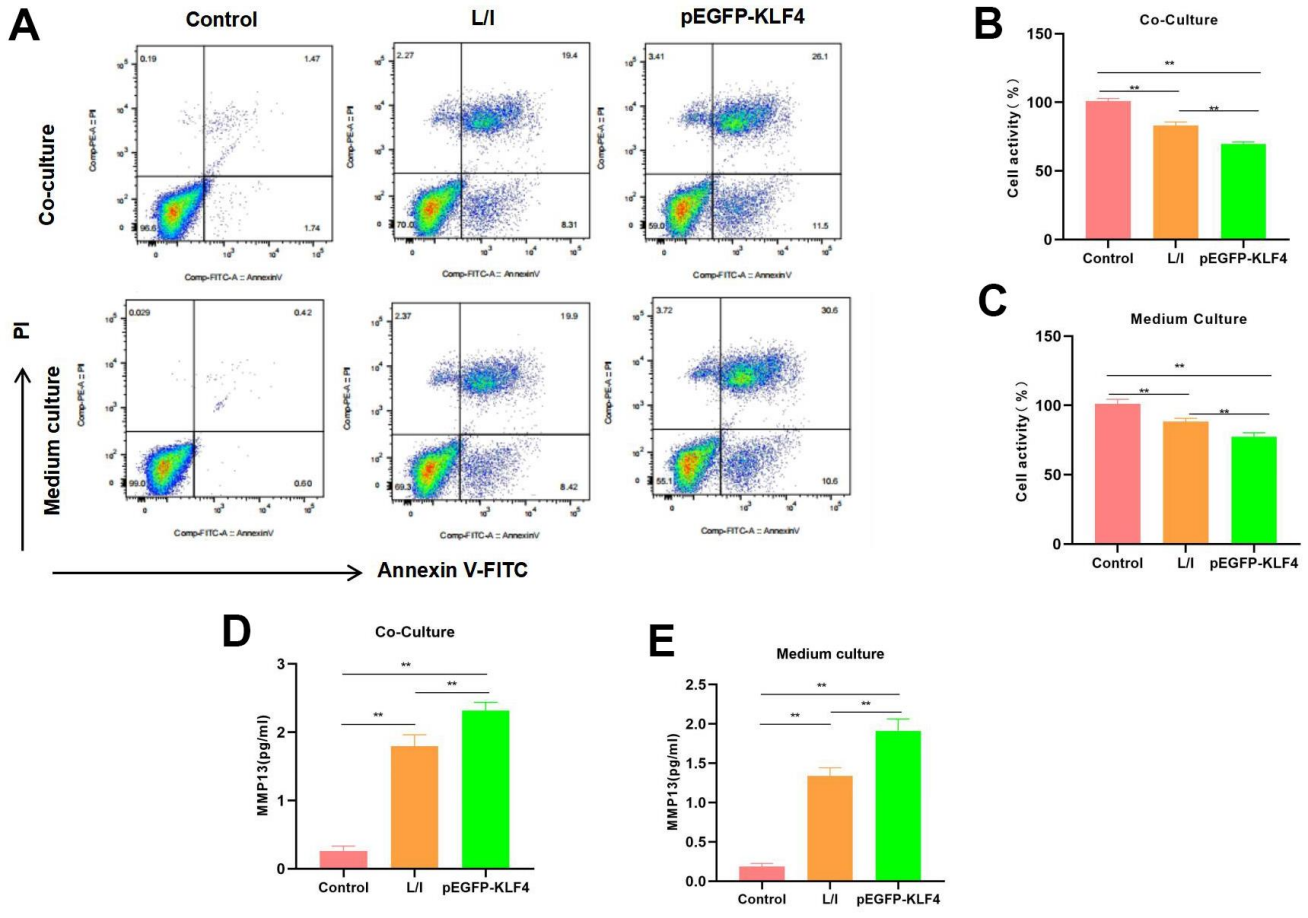
**Effect of M1 macrophages on chondrocyte injury.** (**A**) Flow cytometry (n=3): Chondrocytes co-cultured with M1 macrophages and those cultured with culture medium exhibited obvious apoptosis, macrophages with KLF4 overexpression induced more severe chondrocyte apoptosis than ordinary macrophages, and the apoptosis rate was up-regulated. (**B**, **C**) Cell viability detection (n=3) results suggested that, cells in Control group did not show obvious apoptosis, while chondrocytes co-cultured with M1 macrophage/cultured with culture medium had markedly decreased viability. **P<0.01 between groups. (**D**, **E**) Expression of MMP13 (n=3). The expression level of MMP13 in Control was lower, while in L/I, both co-culture and medium could increase the expression of MMP13 in chondrocytes, After KLF4 overexpression, the level of MMP13 was further increased, with a significant difference compared with L/I, **P<0.01 between groups.

We co-cultured M1 macrophages with chondrocytes and collected M1 cell culture medium to culture chondrocytes. The results suggested that both conditions induced the apoptosis of chondrocytes. As revealed by flow cytometry, macrophages with KLF4 overexpression had a significantly higher cell apoptosis level than that of L/I group, either in the co-culture system or in chondrocytes cultured with culture medium (Figure 4A). Cell viability detection results indicated that, cells in Control group did not show any significant injury and had high viability. Comparatively, the M1 macrophages-induced chondrocyte injury was significantly lower than Control group, while macrophages with KLF4 overexpression induced more severe chondrocyte apoptosis (Figure 4B, 4C). The expression level of MMP13 in Control was lower, while in L/I, both co-culture and medium could increase the expression of MMP13 in chondrocytes, After KLF4 overexpression, the level of MMP13 was further increased, with a significant difference compared with L/I (Figure 4D, 4E).

### Effect of KLF4 on RA mice

**Figure 5:**
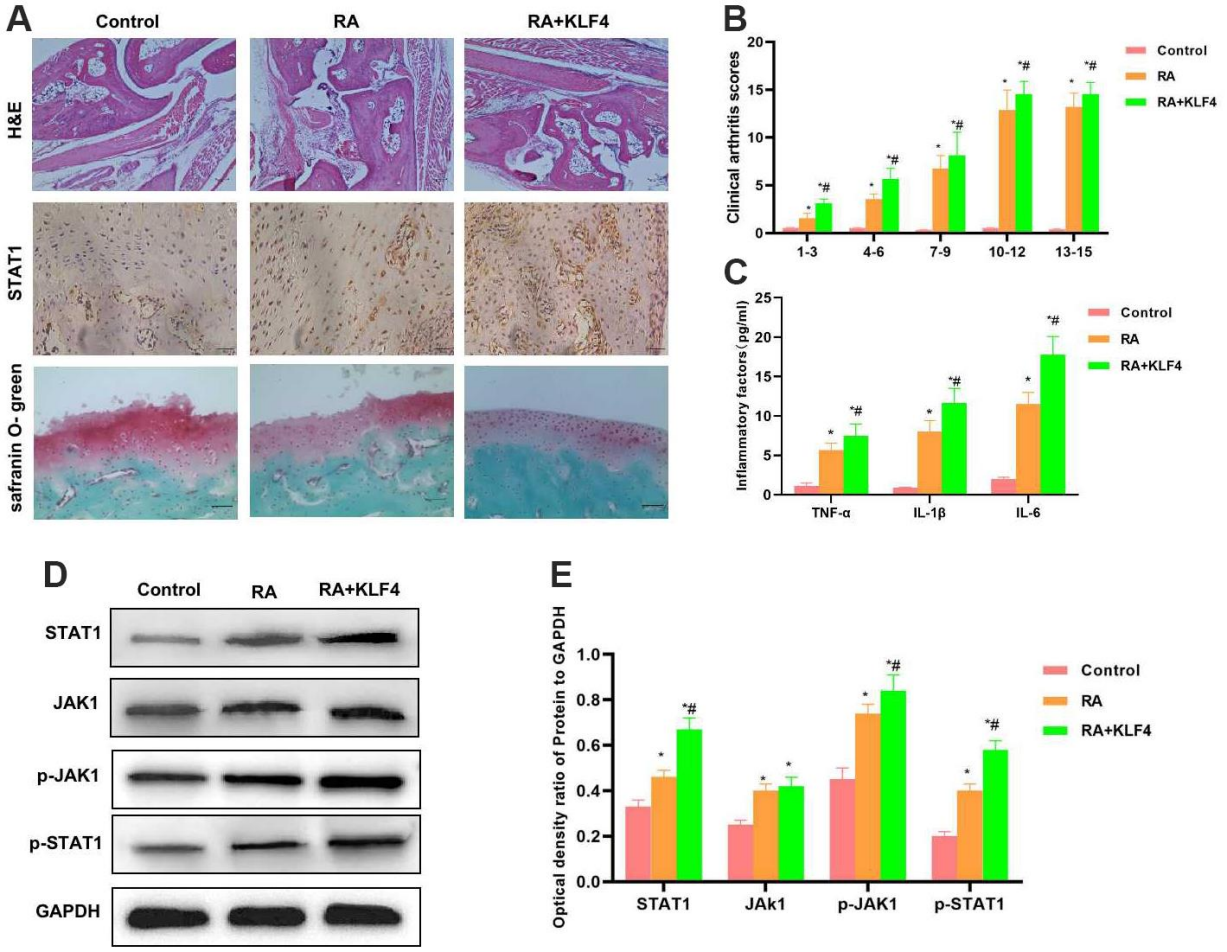
**Effect of KLF4 on the pathology of RA mice (n=3).** (**A**) In Cartilage surface and transitional layer, H&E staining of mouse joint tissues, IHC staining of STAT1 and Safranin O-fast green staining results (n=5). H&E staining results showed that, KLF4 further induced cartilage injury, and obvious joint tissue injury was observed in mice, which was more severe than that of RA group. KLF4 induced STAT1 expression in joint tissues, and the expression in RA+KLF4 group was markedly higher than that in RA group. Safranin O-fast green staining revealed diffuse staining in RA+KLF4 group, which suggested the more severe cartilage injury than that of RA group. (**B**) Mouse clinical pathological score (n=10). RA group had a remarkably higher score than Control group, and RA+KLF4 group had a markedly higher score than RA group. ^*^P<0.05 compared with Control group, ^#^P<0.05 compared with RA group. (**C**) Expression of inflammatory factors in mouse joint tissues (n=5). The inflammatory factor levels in RA group were markedly higher than those in Control group, while those in RA+KLF4 group were further up-regulated, higher than those of RA group. ^*^P<0.05 compared with Control group, ^#^P<0.05 compared with RA group. (**D**, **E**) Expression of JAK1-STAT1 signal proteins (n=5). The JAK1-STAT1 signals in RA group were activated, and their protein and phosphorylation levels were remarkably up-regulated, higher than those of Control group. Meanwhile, the protein levels in RA+KLF4 group were further up-regulated, higher than those of RA group. ^*^P<0.05 compared with Control group, ^#^P<0.05 compared with RA group.

As revealed by H&E staining of mouse joint tissues, cartilage tissues in Control group did not show any obvious injury, with normal structure and with no prominent inflammatory response. By contrast, in RA mice, the cartilage structure was destroyed, and there was tissue inflammatory response. In RA+KLF4 group, more severe cartilage structural destruction than RA group was observed, suggesting that KLF4 overexpression affected the damage of bone and joint. Safranin O-fast green staining results also indicated that, cartilage injury was observed in RA group, with diffuse staining, whereas more severe diffuse staining was observed in RA+KLF4 group. According to IHC staining, STAT1 expression in RA group increased compared with Control group, and that in RA+KLF4 group was higher than that in RA group (Figure 5A). Similarly, clinical pathological score results demonstrated that, RA+KLF4 group had a markedly higher score than RA group, suggesting the more severe RA (Figure 5B). The inflammatory factor levels in RA+KLF4 group were remarkably higher than those in RA group (Figure 5C). Protein detection results indicated that, the JAK1-STAT1 signals in tissues were activated, besides, the levels of JAK1, STAT1, p-JAK1 and p-STAT1 in RA group were markedly higher than those in Control group, and the protein levels were further up-regulated in RA+KLF4 group, evidently higher than those in RA group (Figure 5D, 5E).

## DISCUSSION

Rheumatoid arthritis (RA) is a kind of multi-system inflammatory autoimmune disease that mainly affects bones and joints [11]. Its pathogenic process involves numerous distinct pathways including the innate immune system and adaptive immune system [12]. Although the pathogenic mechanism of RA remains unclear, plenty of studies have suggested that mononuclear cells/macrophages and neutrophils participate in RA genesis and development [13, 14]. Macrophages not only phagocytize and kill pathogenic microorganisms, but also produce multiple pro-inflammatory cytokines and chemokines to participate in the RA pathogenic process [15, 16]. The phenotype and function of macrophages are heterogeneous, which show different phenotypes and functions under the induction of different factors, namely, the M1 and M2 macrophages, which is also referred to as the polarization of macrophages [17]. During the RA disease development process, multiple factors will break the dynamic balance of M1/M2 macrophages induce the imbalance of cell quantity and proportion [18], and cause the continuous increase in M1 pro-inflammatory macrophages, thus aggravating inflammatory response [19]. The polarization of macrophages is regulated by different signaling pathways such as JAK/STAT, PI3K/Akt, JNK and Notch [20, 21]. Molecules such as AKT2, RBP-J, STAT1, p65/p50, p38, NF-κB and AP-1 are mainly related to M1 macrophages [22], whereas molecules like SMAD3, AKT1, STAT3, STAT6, p50/p50 and SMAD2/3/4 are mostly associated with M2 macrophages [23, 24]. Moreover, some signaling molecules are involved in the activation of macrophages, including IPPAR, KLF, IRF, STAT, NF-κB, HIF-1α, HIF-2α, NLRs, GM-CSF, SOCS, phosphatase SHIP, demethylase jmjd3, and peroxidase proliferator-activated receptor-γ (PPAR-γ) [25–27].

STAT1 is one of the important signaling proteins that regulate the M1 polarization of macrophages, but the regulatory mechanism has not been clearly reported yet. KLF4 is a transcription factor, which exerts its effect through regulating mRNA transcription. Previous research has found that KLF4 plays an important role in RA, which is related to the immune balance like Th17. In our study, we introduced KLF4 into macrophages. First of all, we over-expressed KLF4 in macrophages and induced M1 polarization through LPS and IFN-γ. The results discovered that, KLF4 overexpression promoted M1 polarization, increased the expression of JAK1 and STAT1, up-regulated their phosphorylation levels. It is reported that STAT1 is an important signal of polarization [28]. We discovered through RIP assay that, KLF4 bound to STAT1, and speculated that KLF4 exerted its effect through STAT1 transcription. In silencing experiment, KLF4 expression was silenced, which decreased STAT1 expression and suppressed M1 polarization of macrophages. M1 macrophages are mainly a kind of pro-inflammatory cells, their polarization significantly increases the expression of inflammatory factors; in the meantime, inflammatory factors are also the marker cell molecules of M1 macrophages, which have important pro-inflammatory activities. According to our results, pretreatment with STAT1 inhibitor suppressed the effect of KLF4 overexpression, suggesting that STAT1 was the target for the KLF4-mediated signal. As a result, suppressing STAT1 reversed the effect of KLF4 overexpression. By conducting positive feedback experiment, we determined that STAT1 was the target protein of KLF4.

According to our results, the pro-inflammatory effect of macrophages further led to cartilage injury and promoted RA progression. In this regard, we co-cultured M1 macrophages with chondrocytes, and cultured chondrocytes with M1 cell culture medium. Both of the two experiments indicated the apoptosis of chondrocytes, indicating that the KLF4-mediated M1 cells aggravated chondrocyte injury, which was a manifestation of function. Also, we discovered from mouse experiment that, KLF4 overexpression promoted pathologic changes in joint tissues, led to obvious chondrocyte injury and inflammatory response. Moreover, Safranin O-fast green staining results also suggested that, KLF4 overexpression resulted prominent cartilage injury. At the same time, STAT1 protein expression was up-regulated, consistent with cell experimental results.

## CONCLUSIONS

In this study, we discovered that KLF4 promoted M1 polarization of macrophages through STAT1 mRNA transcription, which induced the inflammatory response in RA and further aggravated chondrocyte injury, thus leading to RA progression. KLF4 is promising to be a new therapeutic target for RA.

## MATERIALS AND METHODS

### Induction and intervention of M1 polarization of mouse mononuclear macrophages RAW 264.7

The mouse mononuclear macrophages RAW 267.7 (Procell Biotechnology Co., Ltd., Wuhan, China) were cultured with complete medium under 37° C and 5% CO_2_ conditions. In the overexpression experiment, cells were divided into Control, LPS/IFN-γ (L/I) and pEGFP-KLF4 groups. Among them, the routinely cultured cells were in Control group, whereas cells in the remaining two groups were treated with 100 ng/ml LPS and 30 ng/ml IFN-γ to induce M1 polarization. In the KLF4 silencing experiment, cells were divided into Control, LPS/IFN-γ (L/I) and siRNA-KLF4 groups.

pEGFP-KLF4 was used to construct RAW 267.7 cells with KLF4 overexpression. The detailed transfection method was shown in the following. The eukaryotic plasmid pEGFP-KLF4 (Genepharm Biotechnology Co., Ltd., Shanghai, China) was used for KLF4 overexpression. RAW 267.7 cells were maintained in complete medium at 37° C containing 5% CO_2_ saturated humidity. Cells were passaged every 3-5d. Cells in logarithmic phase were used for transfection. To be specific, cells were seeded into 6-well plates, and transfection was performed when cell confluency reached approximately 80%. Before transfection, serum-containing medium was discarded, cells were washed with PBS for two times, and added 1ml of Opti-MEM medium (Gibco, USA). Afterwards, 1.5μg of plasmid and Lipofectmin2000 were diluted in 100μl Opti-MEM medium (Gibco, USA). After incubation and vortex, the mix was added to cells for incubation. After incubation for 6 h, cells were added with fresh complete medium for further incubation.

(1) Flow cytometry was performed to detect the proportion of F4/80+CD86+M1 cells. In brief, RAW 264.7 cells were subject to LPS/IFN-γ induction for 48 h. Then, the culture medium was collected for ELISA analysis. After cell centrifugation, cells were washed with pre-chilled PBS twice and fixed with pre-chilled methanol, which were incubated with 10 μl FITC-labeled CD86 monoclonal antibody and PE-labeled F4/80 monoclonal antibody (BD, USA) for 20 min in dark. After washing with PBS twice, cells were resuspended with 50 μl solution. Subsequently, the suspension was loaded for detection. The results were expressed as %.

(2) ELISA was performed to detect the expression of M1 macrophage markers. RAW 264.7 cells were subject to LPS/IFN-γ induction for 48 h, the isolated cell medium was preserved at -80° C. After all samples were obtained, they were thawed in the 37° C water bath and centrifuged at 3000 r/min for 20 min. Next, cell markers were detected in line with the ELISA kit (Abcam, USA) instructions. Using the standard curve method, the expression levels of TNF-α, IL-1β and IL-6 were calculated. The results were expressed as pg/ml.

(3) Real-time quantitative PCR (RT-qPCR) was carried out to detect the TNF-α, IL-1β and IL-6 mRNA expression levels. To be specific, total RNA was extracted by employing the Trizol method, and the absorbance (OD) value was detected by ultraviolet spectroscopy to be over 0.8. The primers of TNF-α, IL-1β, IL-6 and GAPDH (internal reference) were provided by Shanghai JEEMA Pharmaceutical Technology Co., Ltd. Primers were designed to span an intron and the sequences were as follows :IL-1β, sense: forward 5′-GCA ATGAGGATGACTTGTTCTTTG-3′ and reverse 5′-CAGAGGTCCAG GTCCTGGAA-3′; TNF-α, sense:5′-ACCTCTCTCTAATCAGCCCTCT-3′ and anti-sense:5′-GGGTTTGCTACAACATG GGCTA; IL-6, sense: 5′-AGCCACTCACCTCTT CAG AAC-3′ and anti-sense: 5′-ACATGTCTC CTTTCTCAGGGC-3′; IL-17, sense: 5′CCCGGACT GTGATGGTCAAC-3′ and anti-sense: 5′GCACTTTGCCTCCCAGATCA3′; β-actin, 5′-CCTGACTGACTACCTCATG AAG-3′ and anti-sense: 5′-GACGTAGCACAGCTT CTCCTTA-3′.

The reverse transcription system consisted of 5xgDNA buffer (2 μl), 10x RT buffer (2 μl), FastKing RT Enzyme Mix (1 μl), FQ-RT Primer Mix (2 μl), and RNA (2 μg), which was diluted with RNase-Free ddH_2_O to 20 μl. The reverse transcription conditions included 42° C for 15 min and 95° C for 3 min. Besides, cDNA was obtained as the template for amplification. The amplification system contained 10xPCR buffer (2.5 μl), dNTPs (2.5 μl), respective primers (0.5 μl each) and cDNA (2 μl), which was diluted with RNase-Free ddH_2_O to 20 μl. The reaction conditions were shown including 95° C for 2 min, 95° C for 30 s, 59° C for 30 s, and 72° C for 30 s for a total of 36 cycles. The 2^-ΔΔCt^ value was calculated by the formula ΔCt=Ct (target gene) - Ct (internal reference gene).

(4) Immunofluorescence (IF) assay was performed to detect the change in CD86 expression. RAW 264.7 cells were subject to LPS/IFN-γ induction for 48 h, washed with PBS thrice, and fixed with 4% formaldehyde for 0.5 h at room temperature. Thereafter, cells were permeabilized with 0.2% Triton X-100 for 5 min, and incubated with CD86 monoclonal antibody (dilution, 1:300; Abcam, USA) at 4° C overnight. After washing with PBS twice, cells were further incubated with fluorescence secondary antibody and observed under the fluorescence microscope after 95% glycerin sealing.

(5) Western blotting (WB) assay was conducted to detect the protein expression levels. After LPS/IFN-γ induction for 24 h, all RAW 264.7 cells were collected, washed with pre-chilled PBS twice, and lysed with 0.5 ml NP-40 lysis buffer on ice for 30 min. After centrifugation, the supernatants were collected to quantify protein contents by the BCA method and adjust protein concentration. Thereafter, proteins were separated by SDS-PAGE and transferred onto PVDF membranes. Afterwards, the membranes were blocked with 5% non-fat milk powder for 2 h, and then incubated with TBST-diluted monoclonal antibodies against JAK1, STAT1, p-JAK1 and p-STAT1 (Abcam, USA). After washing with TBST twice, membranes were further incubated with horse radish peroxidase (HRP)-labeled goat anti-rabbit IgG antibody (dilution, 1:2000; Abcam, USA). After incubation, protein blots were detected by employing the chemiluminescence method, and the OD value was analyzed by Image Pro-Plus 6.0 software, with GAPDH as the internal reference. The results were expressed as the OD ratio of target protein to internal reference protein.

### Verification of the mechanism of KLF4 in promoting the M1 polarization of mononuclear macrophages RAW 264.7 via STAT1

To verify that KLF4 exerted its effect via STAT1, RAW 264.7 cells were divided into L/I, L/I+KLF4, and L/I+KLF4+Cerulomycin groups. Cells in these three groups were treated with LPS/IFN-γ to induce M1 polarization. For cells in L/I+KLF4 and L/I+KLF4+Cerulomycin groups, they were transfected with overexpression plasmid, and those in L/I+KLF4+Cerulomycin group were pretreated with 15 nM STAT1 inhibitor Cerulomycin before M1 polarization induction. Flow cytometry, ELISA and RT-qPCR assays were then conducted according to the above-mentioned detection methods to detect the marker protein and mRNA expression, whereas IF staining was performed to detect CD86 expression. Besides, WB assay was conducted to detect protein expression.

In RNA-binding protein immunoprecipitation (RIP) assay, we detected the binding relationship between STAT1 and KLF4 using the RIP kit (Millipore Corp, Billerica MA, USA). First of all, cells were transfected with KLF4 overexpression plasmid, and induced with LPS and IFN-γ for 24 h. After washing with pre-chilled PBS, cells were lysed with NP-40 lysate, and the lysate was subsequently incubated with antibody to form the coprecipitation. Later, 50 μl KLF4-labeled magnetic beads were added into the suspension and further incubated with 150 μl lysate overnight. Afterwards, the magnetic bead reagent protein complex was collected and digested with proteinase K, and the mRNA was extracted to detect STAT1 by RT-qPCR.

### Effect of M1 RAW 264.7 cells on chondrocytes

To investigate the effect of KLF4-induced M1 polarization of macrophages on chondrocyte injury, we co-cultured the M1 polarization-induced cells with articular chondrocytes, aiming to observe its influence on chondrocytes. In the experiment, we divided RAW 264.7 cells into Control, LPS/IFN-γ (L/I) and pEGFP-KLF4 groups. After induction, cells and culture medium were separated. The co-culture of macrophages with chondrocytes was conducted in Transwell chambers, and the isolated culture medium was used to culture chondrocytes, so as to observe the impacts of macrophages and culture medium on chondrocytes.

(1) CCK-8 assay was conducted to detect the change in chondrocyte viability. Firstly, chondrocytes were co-cultured with macrophages or cultured with macrophage culture medium for 24 h. Then,10 μl CCK-8 reagent was added for staining. After incubation for 4 h, the OD value was detected at 450 nm with the microplate reader, with blank culture medium as the control. Finally, cell viability was calculated. The results were expressed as %.

(2) Flow cytometry was conducted to detect cell apoptosis level. After chondrocytes were co-cultured for 24 h, all cells were collected, washed with pre-chilled PBS, and centrifuged at 3000 rpm/min for 30 min. After suspension with Binding Buffer, cells were stained, incubated with 5 μl Annexin V-FITC in dark for 5 min and subsequently with 5 μl PI in dark for another 5 min using the cell apoptosis detection kit (BD, USA). After washing with PBS, cells were loaded for detection, and apoptotic cells were calculated as Annexin V-FITC (+) PI (+) and Annexin V-FITC (+) PI (-) cells.

(3) ELISA was performed to detect MMP13: After chondrocytes were co-cultured for 24 h, the isolated cell medium was preserved at -80° C. After all samples were obtained, they were thawed in the 37° C water bath and centrifuged at 3000 r/min for 20 min. Next, cell markers were detected in line with the ELISA kit (Abcam, USA) instructions. The results were expressed as pg/ml.

### Effect of KLF4 on RA mice

A total of 30 8-10-week-old BALB/c mice were randomly divided into Control, RA and RA+KLF4 groups. To construct the RA model, collagen II monoclonal antibody complex and lipopolysaccharide (LPS) were intraperitoneally injected into mice to induce RA formation. In brief, the collagen II monoclonal antibody complex (5 mg/kg/d) was intraperitoneally injected into mice for 10 consecutive days. On days 1 and 4 of intraperitoneal injection of collagen II monoclonal antibody complex, each mouse was injected with 100 μg LPS. Meanwhile, the stable collagen antibody-induced RA model was constructed in about two weeks. Mice in RA+KLF4 group were given intraarticular injection of KLF4 overexpression plasmid liposome every three days.

(1) Measurement of arthritis score: Arthritis scores were measured for 5 times during the 10-day modeling and 5-day further culture process at intervals of 3 days. The arthritis in mice was assessed using the macroscopic scoring system. The standards are as follows, 11-15 points: the entire foot claws and toes exhibit severe arthritis; 6-10 points: more than two joints show severe arthritis; 1-5 points: two joints show inflammatory manifestation; and 0 point: no arthritis manifestation.

(2) ELISA was performed to detect inflammatory factors in peripheral blood. After modeling, mice were further fed for 5 days. Later, the peripheral blood samples were collected from the mouse posterior orbital vein, treated with heparin for anticoagulation, and centrifuged at 6000 rpm for 5 min to collect the serum. Finally, the expression of TNF-α, IL-1β and IL-6 was detected in line with the above-mentioned method.

(3) H&E staining of mouse joint tissues was conducted to detect pathologic changes. The joint tissues were fixed with 4% paraformaldehyde (PFA), dehydrated, permeabilized, embedded in paraffin, fixed in the wax block, and prepared into sections. Thereafter, the paraffin sections were subject to deparaffinage, hydration, staining, blue staining, dehydration, permeabilization and sealing in succession according to the routine HE staining steps. Images were taken by a microscope.

(4) Immunohistochemistry (IHC) was carried out to detect STAT1 expression. Firstly, joint tissues were fixed with 4% PFA, embedded in paraffin and sliced into serial sections. Then, the tissue sections were soaked in the 1:50 acetone solution for 3 min, dried in the air, soaked in xylene, and treated with gradient ethanol concentrations. Thereafter, sections were exposed to 0.01 mol/L citric acid buffer for antigen retrieval, treated with 3% hydrogen peroxide for 10-15 min to eliminate the endogenous peroxidase, blocked with 5% BSA, and treated for 15-30 min at 37° C. Afterwards, sections were incubated with STAT1 monoclonal antibody (Abcam, USA) at 37° C, further incubated with secondary antibody, and stained with DAB color development agent. After hematoxylin counter-staining, sections were soaked with gradient ethanol concentrations, dehydrated, permeabilized and sealed with resin.

(5) Safranin O-fast green staining. The joint tissue paraffin sections were deparaffinized to hydration. To be specific, the sections were soaked with xylene I for 20 min, xylene II for 20 min, absolute ethyl alcohol I for 5 min, absolute ethyl alcohol II for 5 min, and 75% ethanol for 5 min, and then washed with tap water. In fast green staining, sections were soaked in fast green solution for 5-10 min, the redundant dye was removed by water washing until the cartilage became colorless. Later, the cartilage was soaked in the differentiation solution and washed with tap water. In safranin O staining, the sections were soaked in safranin O solution for 15-30 s, and rapidly dehydrated with three pumps of absolute ethyl alcohol. The sections were permeabilized with clean xylene for 5 min, and mounted with neutral resin. Finally, the sections were observed under a microscope. Besides, images were collected and analyzed.

(6) WB assay was conducted to detect the expression of key proteins. The RIPA lysate was utilized for cell lysis and protein extraction. Subsequently, the protein content was quantified by the BCA kit. The protein expression of JAK1, STAT1, p-JAK1 and p-STAT1 was detected according to the above description.

### Statistical analysis

The SPSS19.0 software was employed for performing statistical analysis. Measurement data were compared by t-test, analysis of variance (ANOVA) and Mann-Whitney U test, whereas log-rank test was adopted for comparison among groups. Measurement data were expressed as mean ± standard deviation $\left(\bar{x}\pm s\right)$ and compared by one-way ANOVA among multiple groups, while by SNK test between two groups. P<0.05 stood for statistical significance.

### Data availability statement

The data that support the findings of this study are available from the corresponding author upon reasonable request.

### Ethical approval and consent to participate

The study approved with Ethics Committee.

### Consent for publication

All authors approval published the article.
